# Differential effects of weather, plant phenology and predators on the seasonal variation of aphids on cabbage

**DOI:** 10.1111/jen.13106

**Published:** 2023-02-06

**Authors:** Ethelyn Echep Forchibe, Ken Okwae Fening, Benjamin Narh‐Madey, Kwame Afreh‐Nuamah, Millicent Asaaba Cobblah, Francis Onono Wamonje, John Peter Carr

**Affiliations:** ^1^ African Regional Postgraduate Programme in Insect Science (ARPPIS), College of Basic and Applied Sciences University of Ghana Accra Ghana; ^2^ Soil and Irrigation Research Centre, School of Agriculture, College of Basic and Applied Sciences University of Ghana Accra Ghana; ^3^ Department of Crop Science, School of Agriculture, College of Basic and Applied Sciences University of Ghana Accra Ghana; ^4^ Department of Animal Biology and Conservation Science, School of Biological Sciences, College of Basic and Applied Sciences University of Ghana Accra Ghana; ^5^ Pest and Pathogen Ecology National Institute of Agricultural Botany (NIAB) East Malling UK; ^6^ Department of Plant Sciences University of Cambridge Cambridge UK

**Keywords:** agroecological zone, coccinellids, *Lipaphis erysimi pseudobrassicae*, *Myzus persicae*, spiders, syrphids

## Abstract

The aphids *Lipaphis erysimi pseudobrassicae* (Davis) and *Myzus persicae* (Sulzer) pose serious threats to the production of cruciferous crops in the tropics. Understanding their population dynamics is important for developing integrated pest management programmes to minimize their damage to crops. This study investigated the effects of climatic factors, natural enemies and plant age on the population dynamics of these pests. The population density of aphids and their natural enemies in 20 cabbage plants, and weather conditions were monitored for five cropping seasons from 2019 to 2021 in two agroecological zones of Ghana (Coastal Savannah and Deciduous Forest zones). The highest population density of *L. e pseudobrassicae* was recorded in January (dry season) in both agroecological zones, while the highest population density for *M. persicae* occurred in September (minor rainy season) and August (dry spell) in the Coastal Savannah and Deciduous Forest zones, respectively. The highest aphid densities were noted to occur during periods with low relative humidity and low rainfall. The population density of *L. e. pseudobrassicae* was significantly negatively related to plant age, air temperature and relative humidity, and positively related to syrphids (*Paragus borbonicus*) and spiders in the Coastal Savannah zone, while in the Deciduous Forest zone, it was significantly positively related to coccinellids. On the other hand, *M. persicae* population density was significantly positively related to syrphids and coccinellids in the Deciduous Forest zone. Rainfall negatively affected syrphids in the Coastal Savannah zone, while air temperature positively affected syrphids and negatively affected spiders in the Deciduous Forest zone. Coccinellids had a significant positive relationship with relative humidity in the Deciduous Forest zone. This study provides important insights into the key factors that regulate aphid population densities on cabbage and will support development of timely interventions to manage these pests.

## 1 INTRODUCTION

The aphids *Lipaphis erysimi pseudobrassicae* (Davis) and *Myzus persicae* (Sulzer) are serious pests of cabbage and other brassica crops cultivated in Ghana and around the world (Liu & Yue, 2000; Forchibe, Fening, & Afreh‐Nuamah, 2017; Fening et al., 2020; Adenka, Fening, Afreh‐Nuamah, Wamonje, & Carr, 2021). After infestation by aphids, plants often deteriorate faster due to high reproductive rates which leads to a rapid population build‐up (Dixon, 1977). Indeed, severe infestations can cause up to 100% crop loss, especially on young plants, and they are also major vectors of plant viruses (Rana, 2005; Razaq et al., 2011; Yarou et al., 2017; Adenka, Fening, Afreh‐Nuamah, Wamonje, & Carr, 2021). While *L. e. pseudobrassicae* is a specialist feeder on crucifers, *M. persicae* is a generalist feeder, feeding on host plants in over 40 plant families (Blackman & Eastop, 2000, 2017).

Aphids pose a major threat to vegetable production because of their rapid evolution of resistance to available insecticides (Ntow, Gijzen, Kelderman, & Drechsel, 2006; Bass et al., 2014); thus, an integrated pest management (IPM) approach is needed. Developing IPM approaches to pest requires a thorough understanding of a pest's biology and ecology and its interaction with the environment (Fidelis et al., 2018; Dent & Binks, 2020). For example, the variation in the abundance of aphids on crops is influenced mainly by: climate, inter‐specific and intra‐specific abundance of other insects, and oftentimes plant phenology (Agarwala & Datta, 1999; Van Emden & Bashford, 1971). These factors can cause direct or indirect changes to aphids' fertility, mortality and migration rates (Fidelis et al., 2019). In addition, abiotic factors such as rainfall, temperature, and relative humidity, and biotic factors such as the incidence of natural enemies and host plants, all affect the rates at which aphid populations increase and the eventual density of aphids on their hosts (Bale et al., 2002; Nietschke, Magarey, Borchert, Calvin, & Jones, 2007; Soares et al., 2020).

Understanding the population dynamics of pests, including the effects of the environment, allows for establishment of critical sampling periods, treatment thresholds and the adoption and implementation of management strategies. Implementing a timely management strategy prevents pest build‐up beyond economically damaging thresholds and minimizes the overuse of insecticides (Barzman et al., 2015; Dara, 2019). This study sought to understand the incidence and seasonal variation of *L. e. pseudobrassicae* and *M. persicae* on cabbage in relation to climatic and some biotic factors across different cropping seasons.

## 2 MATERIALS AND METHODS

### 2.1 Study sites and period of data collection

This study was carried out from 2019 to 2021 in Kpong (Lat. 6.134874, Long. 0.08207, Alt. 23 m) and Aseseeso (Lat. 6.009533, Long. ‐0.0443213, Alt. 196 m), which are both in the Eastern region of Ghana and are, respectively, located in the Coastal Savannah and Deciduous Forest agroecological zones. Kpong is characterized by an annual rainfall of between 700 and 1100 mm, an average annual temperature of 28°C and relative humidity between 59% and 93%. Asseseeso is characterized by an annual rainfall of between 900 and 1270 mm, an average annual temperature of 25°C, and relative humidity between 72% and 95%. Both locations have two seasons with a bimodal rainfall pattern which includes a major rainy season (from April to July), a minor rainy season (from September to November) and a dry season (from December to March) (MoFA (Ministry of Food and Agriculture), 2020). The Deciduous Forest agroecological zone is characterized by a warm and moist climate, frequent rainfall, high vegetative diversity and very fertile soils. In contrast, the Coastal Savannah agroecological zone is characterized by warm temperatures all year round with fertile soils and fewer rainfall days (Kemausuor, Akowuah, & Ofori, 2013). The study was carried out for five cropping seasons within 2 years, from 22 August 2019 to 2 February 2021 in the Coastal Savannah agroecological zone and from 22 July 2019 to 10 February 2021 in the Deciduous Forest agroecological zone (Table 1).

**TABLE 1:** Cropping seasons/study periods.

Season	Study period		Seasons
	Coastal Savannah	Deciduous Forest	
Cropping season 1	22 August to 24 October 2019	22 July to 23 September 2019	Minor rainy season
Cropping season 2	20 November 2019 to 14 January 2020	12 November 2019 to 7 January 2020	Minor rainy to dry season
Cropping season 3	19 March to 21 May	11 March to 7 June	Major rainy season
Cropping season 4	1 September to 2 November	29 September to 30 November	Minor rainy season
Cropping season 5	1 December 2020 to 2 February 2021	9 December 2020 to 10 February 2021	Dry season

### 2.2 Land preparation, nursery establishment and transplanting

Experimental plots were cleared of weeds, ploughed and ridged 1 week after nursery establishment. The soil was mixed with decomposed poultry manure (20 t/ha) and left to fallow for 2 weeks before transplanting of ‘hardened‐off’ cabbage seedlings. Plots were demarcated, with each measuring 1 m × 5 m. Seeds of disease‐free certified healthy hybrid white cabbage (*Brassica oleracea* var. *capitata*) cv. Oxylus were purchased from AGRI‐SEED Limited, Accra, Ghana and germinated in trays containing a mixture of soil and Gro‐Plenty organic compost (Green‐Gro, Ghana). Oxylus was chosen because it is the most preferred cabbage variety by farmers due to its compact head and longer shelf life (Amoabeng et al., 2017). Trays were kept in a screen house to prevent attack by insect pests. Weeding, thinning and irrigation were done regularly as needed. Seedlings were transplanted 4 weeks after germination (having four to six true leaves) at a planting distance of 50 cm × 50 cm onto demarcated plots. No insecticide was applied during the growth of the plants.

### 2.3 Sampling for aphids and predators

The population density of *L. e. pseudobrassicae, M. persicae* and their associated natural enemies was evaluated at 7‐day intervals, from the crop's 5 to 6 true‐leaf stage (2 weeks after transplanting) to the head fill stage (9th to 10th week) (Andaloro, Rose, Shelton, Hoy, & Becker, 1983) throughout all the cropping seasons. Sampling for aphids was based on methods from Hughes (1963) and Forchibe, Fening, and Afreh‐Nuamah (2017), which involved destructive sampling of leaves of cabbage plants. Despite, some perceived shortcomings of this method, it is one of the most efficient (Hughes, 1963; Schmidt, & O'Neal, M. E., & Dixon, P. M., 2008) to offer detailed information on the actual insect counts, as it is practically impossible to count large insect numbers, if you are using non‐destructive sampling methods such as rapid visual estimates. Thus, the sampling was carried out in such a way that plants were selected from different parts of the field, hence allowing insect population to recover quickly. The third, fourth and fifth expanded leaves were removed from each randomly sampled 20 cabbage plants and placed in a 30 cm × 20 cm × 10 cm bowl during each sampling date. In the laboratory, aphids, parasitized forms (mummies) as well as natural enemies were counted and recorded. Parasitized aphids were reared for emergence of the parasitoid (*Aphelinus varipes* Förster) and were only recorded in very low numbers for the last 3 weeks of the first cropping season. Data recorded on the parasitoid were inadequate, and thus not included in the analysis.

### 2.4 Identification of insects

Aphids were identified following morphological keys by Blackman and Eastop (2000) and confirmed by molecular methods described in Fening et al. (2020). Coccinellids were identified by comparing with reference specimens deposited at the Insect Museum of the Department of Animal Biology and Conservation Science (DABCS), University of Ghana (Fening, Tegbe, & Adama, 2014). Syrphids were identified following keys by Stuckenberg (1954) and Vujić, Ståhls, Rojo, Radenković, and Šimić (2008) by a curator; H. Davies at the Insect Museum of the DABCS. Samples of larvae of both syrphids and coccinellids were cultured in the laboratory to the adult stage to allow for identification. Parasitoids were identified using molecular method (DNA Barcoding) (Folmer, Black, Hoeh, Lutz, & Vrijenhoek, 1994).

### 2.5 Weather data

Weather data including average daily air temperature, rainfall and relative humidity (RH) during the study period were obtained from the University of Ghana Soil and Irrigation Research Centre weather station, Kpong and the National Ghana Meteorological Agency. The weekly mean values of air temperature, RH and 2 weeks cumulative rainfall prior to each evaluation time were used in the statistical analysis.

### 2.6 Statistical analysis

Statistical analyses were performed in R 4.2.1 (R Core Team, 2022). The relationship between population of *M. persicae*, and *L. e. pseudobrassicae* as the dependent variables and plant age, air temperature, relative humidity and rainfall as the explanatory variables were studied. The predators were also studied against these weather variables and aphid densities. After determining overdispersion in the exploratory analysis, using generalized linear mixed models (GLLM) with Poisson distribution and log‐link function (“glmer,” package “lme4”) (Bates, Mächler, Bolker, & Walker, 2015) where the estimated dispersion was greater than one, hurdle models were employed (Mayer, Roy, Robins, Halliday, & Sellin, 2005; Hu, Pavlicova, & Nunes, 2011). Hurdle models better account for both overdispersion and the occurrence of zero values than can be handled in typical Poisson distribution‐based models. The model is of two parts: a truncated negative binomial, appropriate for positive counts and a hurdle component for handling zeros and larger counts (Mullahy, 1986). As the random variable (sampling time) was not significant, fixed effects truncated negative binomial generalized linear model (GLM) with log‐link function (“pscl,” package “hurdle”) (Cameron & Trivedi, 2005) was adopted. Results showed an increase in the log‐likelihood with associated Wald test, improving the model as compared to the Poisson model (not shown) (Weerahandi & Yu, 2020).

## 3 RESULTS

### 3.1 Abundance of aphids and predators

The most abundant aphid species was *Lipaphis erysimi pseudobrassicae* (Davis), compared to *Myzus persicae* (Sulzer) with a relative abundance of 90.4% and 9.6%, respectively, in the Coastal Savannah agroecological zone and 68.1% and 31.9%, respectively, in the Deciduous Forest agroecological zone. The densities of *M. persicae* varied significantly between the two agroecological zones (t (59) = 2.39, *p* = 0.020). The major predators recorded were Syrphidae (*Paragus borbonicus* Macquart), Coccinellidae (*Cheilomenes lunata* Fabricius*, Cheilomenes propinqua vicina* Mulsant) and spiders. Syrphids were the most abundant predators in both the Coastal Savannah and Deciduous Forest agroecological zones (49.71% and 48.0%) followed by spiders at 40.6% and 43.1%, respectively. Coccinellids were least abundant at 9.07% and 8.9% for the Coastal Savannah and the Deciduous Forest zones, respectively.

### 3.2 Seasonal variation of aphids and predators

**FIGURE 1:**
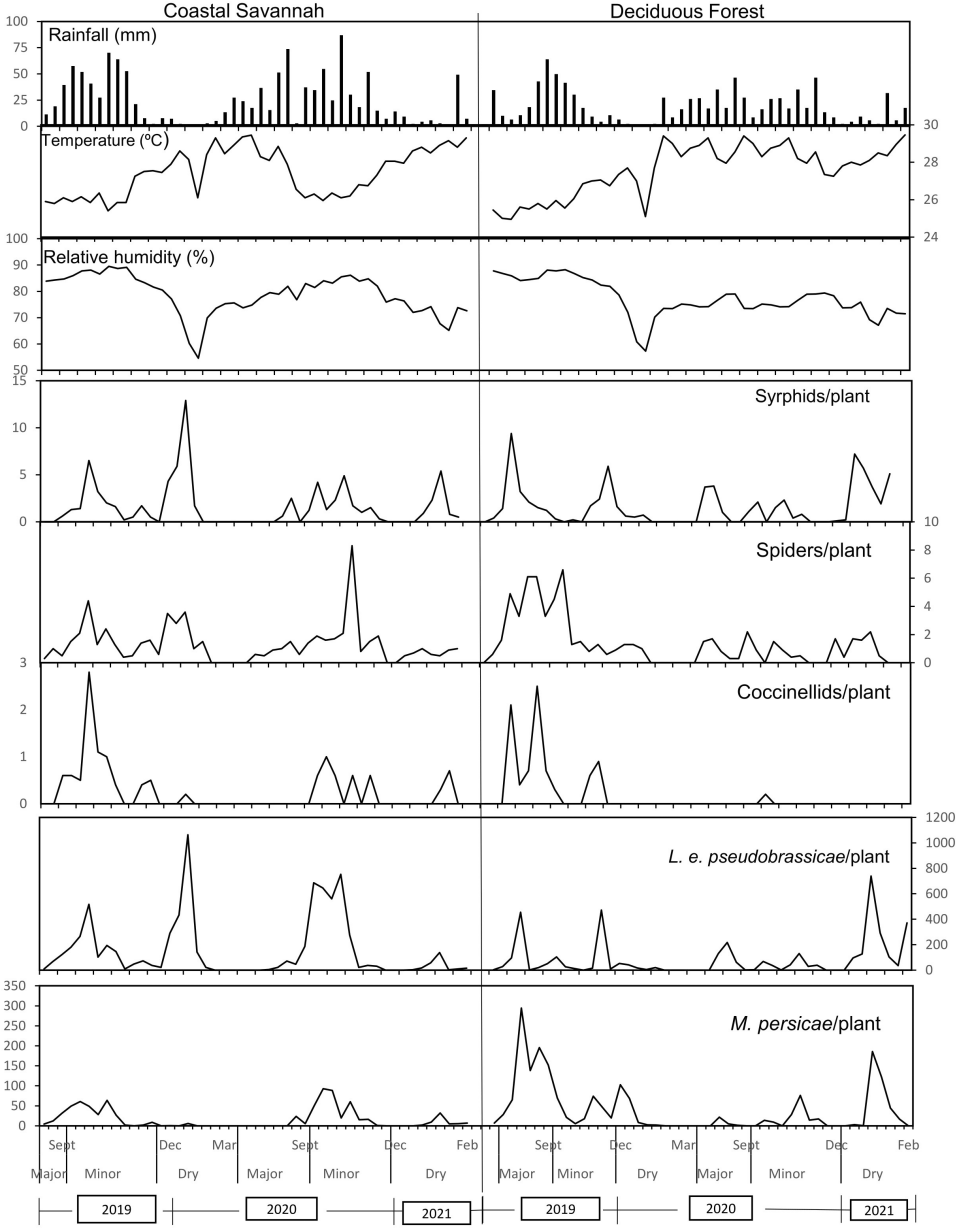
Weather factors and predator/aphid densities on cabbage in the Coastal Savannah and Deciduous Forest agroecological/climatic zones in Ghana. Values shown are the average number of individuals recorded on 20 plants (Major = Major rainy season, Minor = Minor rainy season and Dry = Dry season).

In the Coastal Savannah agroecological zone, the highest density of *L. e. pseudobrassicae* (1063 aphids/plant) was observed in January (dry season) of 2020. Other peaks (600–750 aphids/plant) were recorded in September and October (minor rainy season) of 2020 (Figure 1). In the Deciduous Forest zone, the highest densities (739 aphids/plant) were recorded in January (dry season) of 2021 and other peaks (~470 aphids/plant) were recorded in November (dry season) of 2019. On the other hand, *M. persicae* had very low population densities throughout the study period, with the highest densities recorded in September (minor rainy season) of 2020 in the Coastal Savannah zone (93 aphids/plant), and in August (dry spell) of 2019 in the Deciduous Forest zone (294 aphids/plant). The highest population peaks of both aphids were observed to occur during periods of drought, high temperatures and lower relative humidity.

Syrphids were recorded in high densities in January (~7–12 larvae/plant) (dry season) of 2020 in both agroecological zones (Figure 1). Peaks of spider density appeared irregularly throughout the study period in both zones. Coccinellids were generally low throughout the evaluation period, except in August (major season) and September (minor season) (~2–3 individuals/plant) of 2019 in both agroecological zones.

**FIGURE 2:**
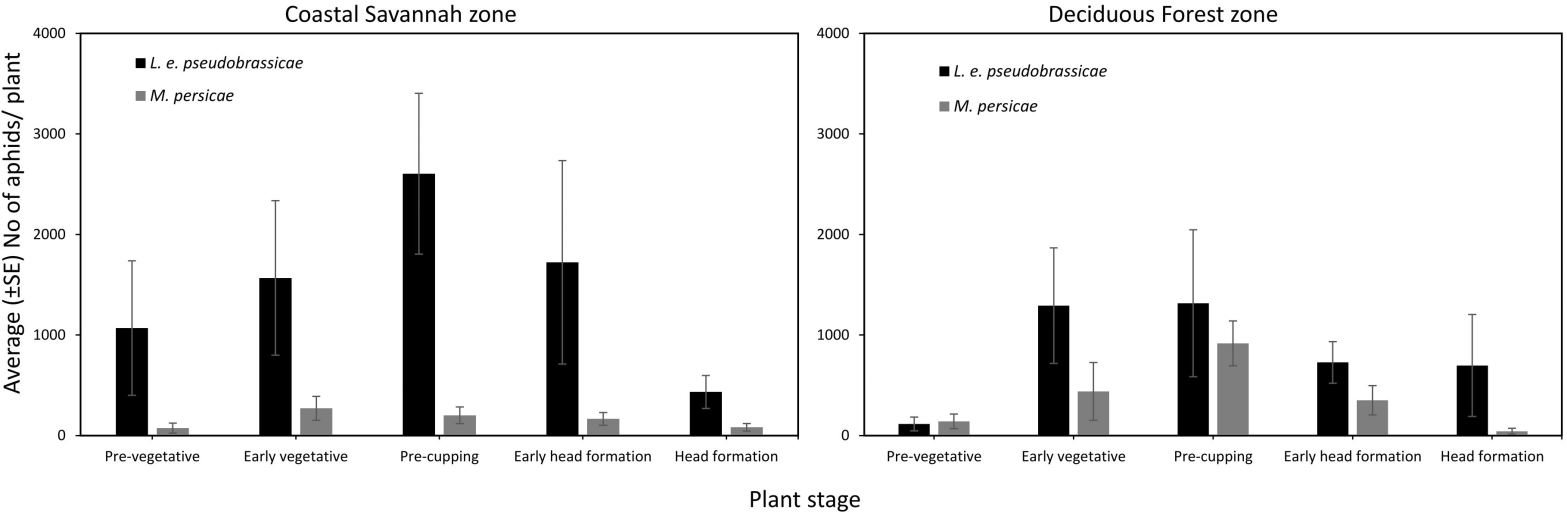
Average (±SE) number of aphids per plant on the different cabbage growth stages in the Coastal Savannah and Deciduous Forest agroecological zones

*Lipaphis erysimi pseudobrassicae* was most abundant on all plant stages in both agroecological zones, except the pre‐vegetative stage in the Deciduous Forest zone (Figure 2). The plant stages with the highest *L. e. pseudobrassicae* infestation (critical stage) were the pre‐cupping stage in the Coastal Savannah zone (~2600 aphids/plant), and the early vegetative (~1200 aphids/plant) and pre‐cupping stages (~1300 aphids/plant) in the Deciduous Forest zone (Figure 2). Populations of *M. persicae* were generally low (~300 aphids/plant) on all plant stages in the Coastal Savannah zone, while in the Deciduous Forest zone the pre‐cupping stage was the critical stage (~ 900 aphids/plant) with the highest *M. persicae* infestation (Figure 2). A significant difference was recorded in the number of *M. persicae* between the different plant stages (df _(4, 42)_ = 3.14, *p* = 0.024), with the pre‐cupping stage recording the highest infestation in the Deciduous Forest agroecological zone.

### 3.3 Factors affecting aphid abundance

The air temperature, RH and plant phenology showed a significantly negative relationship with the density of *L. e. pseudobrassicae*, and rainfall had a significantly positive relationship with *L. e. pseudobrassicae* (*p* = 0.004) in the Coastal Savannah agroecological zone (Table 2). In addition, syrphids (*p* < 0.001) and spiders (*p* = 0.029) were significantly positively related to *L. e. pseudobrassicae* densities in the Coastal Savannah agroecological zone, while syrphids were significantly positively related (*p* < 0.001) to *M. persicae* density in the Deciduous Forest agroecological zone (Table 3).

**TABLE 2:** Summary of Hurdle model with truncated negative binomial distribution comparing weather records and plant age affecting the densities of aphids on cabbage.

Term	Coastal Savannah zone				Deciduous Forest zone			
	Estimate	SE	Z‐value	*p*	Estimate	SE	Z‐value	*p*
*L. e. pseudobrassicae*								
Intercept	26.251	5.419	4.844	**< 0.001**	36.288	32.552	1.115	0.265
Plant age	−0.214	0.094	−2.287	**0.022**	0.249	0.181	1.375	0.169
Air temperature	−0.333	0.096	−3.487	**< 0.001**	−1.132	0.768	−1.475	0.14
Rainfall	0.029	0.010	2.879	**0.004**	0.031	0.050	0.612	0.54
Relative humidity	−0.114	0.037	−3.039	**0.002**	−0.069	0.168	−0.416	0.678
*M. persicae*								
Intercept	8.868	7.517	1.18	0.238	20.864	13.691	1.524	0.128
Plant age	−0.039	0.085	−0.467	0.640	−0.194	0.187	−1.037	0.3
Air temperature	−0.119	0.130	−0.915	0.360	−0.364	0.330	−1.103	0.27
Rainfall	0.022	0.012	1.764	0.078	0.014	0.021	0.676	0.499
Relative humidity	−0.007	0.053	−0.133	0.894	−0.045	0.071	−0.636	0.524

*Note*: Statistically significant *p*‐values are boldfaced.

**TABLE 3:** Summary of the Hurdle model with truncated negative binomial distribution comparing aphid densities and climatic factors affecting the abundance of predators on cabbage.

Term	Coastal Savannah zone				Deciduous Forest zone			
	Estimate	SE	Z‐value	*p*	Estimate	SE	Z‐value	*p*
Syrphid								
Intercept	0.234	3.37	0.695	0.487	−8.940	4.66	−1.92	0.055
*L. e. pseudobrasssicae*	0.00	0.00	3.64	**<0.001**	0.00	0.00	1.14	0.254
*M. persicae*	0.00	0.00	1.284	0.199	0.00	0.00	4.058	**<0.001**
Air temperature	0.037	0.059	0.626	0.531	0.373	0.132	2.82	**0.004**
Rainfall	−0.014	0.006	−2.274	**0.023**	−0.005	0.009	−0.559	0.576
Relative humidity	−0.006	0.024	−0.272	0.785	0.014	0.024	0.598	0.549
Spiders								
Intercept	−2.960	2.830	−1.049	0.294	10.40	3.530	2.949	**0.003**
*L. e. pseudobrasssicae*	0.00	0.00	2.172	**0.029**	0.00	0.00	0.331	0.74
*M. persicae*	0.00	0.00	0.58	0.561	0.00	0.00	1.252	0.21
Air temperature	0.097	0.050	1.925	0.054	−0.294	0.099	−2.962	**0.003**
Rainfall	−0.010	0.005	−1.867	0.062	0.018	0.007	2.393	**0.017**
Relative humidity	0.037	0.020	1.85	0.064	−0.002	0.018	−0.096	0.923
Coccinellids								
Intercept	−26.490	19.090	−1.387	0.165	−29.210	16.180	−1.805	0.071
*L. e. pseudobrasssicae*	0.00	0.00	0.664	0.507	0.00	0.00	2.321	**0.020**
*M. persicae*	0.00	0.00	1.602	0.109	0.001	0.00	2.370	**0.018**
Air temperature	0.554	0.487	1.137	0.256	0.544	0.360	1.511	0.131
Rainfall	−0.003	0.099	−0.333	0.739	0.003	0.009	0.371	0.711
Relative humidity	0.147	0.077	1.901	0.057	0.189	0.083	2.268	**0.023**

*Note*: Statistically significant *p*‐values are boldfaced.

### 3.4 Factors affecting predator abundance

The densities of coccinellids were positively related to RH (*p* = 0.023), and that of both aphid species in the Deciduous Forest agroecological zone, while syrphid density had a significantly negative relationship (*p* = 0.023) with rainfall in the Coastal Savannah zone, and spiders had a positive relationship (*p* = 0.017) with rainfall in the Deciduous Forest zone (Table 3). The abundance of syrphids was dependent on the presence of aphids in both agroecological zones.

## 4 DISCUSSION

Understanding the incidence of a pest and its relationship with biotic and abiotic components is vital, because it allows for the development of effective sampling plans, environmental manipulation and implementation of pest management strategies (Ehi‐Eromosele, Nwinyi, & Ajani, 2013). Furthermore, implementing control strategies during critical periods of pest infestation can curtail unnecessary usage of pesticides (Fidelis et al., 2019).

Different seasonal variations were recorded for *L. e. pseudobrassicae* and *M. persicae* throughout the different seasons and cabbage growth stages with frequent population peaks, and in some seasons, they co‐existed on the field. Although both aphids are known to feed on the middle and lower leaves of brassica plants (Sampaio et al., 2017; Fidelis et al., 2019), their colonization varies depending on the plant stage in both agroecological zones, with *L. e. pseudobrassicae* preferably found in higher densities on all the plant stages compared to *M. persicae*. Furthermore, aphids on cabbage usually present varying population densities, with frequent peaks across different seasons (Singh & Lal, 2012; Prasad, 2003; Fidelis et al., 2019), suggesting that efficient control of these pests is contingent on frequent monitoring throughout each growing season, and not just reliance on calendar spray regimes.

*Lipaphis erysimi pseudobrassicae* population incidence showed a negative relationship with plant age in the Coastal Savannah agroecological zone. Previous studies have shown that plant phenology can affect the population dynamics of aphids (Jansson & Smilowitz, 1985; Pelletier, Pompon, Dexter, & Quiring, 2010), owing particularly to the nitrogen and amino acid contents of host plants (Karley, Douglas, & Parker, 2002; Agarwala, Das, & Rosenheim, 2012). With increasing plant age, leaves showed a decrease in nitrogen content and qualitative changes in amino acid composition, which were detrimental to the growth of aphids (Leite, Picanço, Jham, & Moreira, 2005; Agarwala, Das, & Rosenheim, 2012; Munthali & Tshegofatso, 2014). Furthermore, this study showed decreased aphid population densities during the head formation stage (final growth stage) of cabbage growth, which could probably be directly related to decreased nitrogen content in plants. Thus, perhaps the decreased nitrogen in the older cabbage plant did not favour *L. e. pseudobrassicae* although this was not investigated in the current study. This validates the importance of plant phenology in pest management because susceptibility to pest infestation is often dependent on plant phenology, and as such, management measures must be targeted and employed accordingly (Andaloro, Rose, Shelton, Hoy, & Becker, 1983).

In the Coastal Savannah zone, air temperature and RH negatively affected the density of *L. e. pseudobrassicae*, while rainfall positively affected this aphid. Weather parameters play an important role in the growth, development, distribution and population dynamics of aphids (Bale et al., 2002; Qaisar et al., 2014; Skendžić, Zovko, Živković, Lešić, & Lemić, 2021). In studies by Rao et al. (2013), temperature was found to be the major factor regulating aphid population on brassica plants. Temperatures recorded during the study in this zone ranged from 23.8°C to 31.1°C, which have been reported to be optimal for high reproduction of *L. e. pseudobrassicae* (Liu & Yue, 2000). None of the weather variables affected *M. persicae* in both agroecological zones, possibly due to this highly cosmopolitan aphid's ability to quickly acclimate to local climate conditions (Alford, Blackburn, & Bale, 2012). Furthermore, there was minimal variation in the weather conditions in the Deciduous Forest zone, probably accounting for the non‐relationship recorded between these parameters and the recorded aphid densities. Species abundance is often affected by agroecological barriers, which are usually characterized by differences in physical environments (Shukla, 1990; Faheem, Saeed, Sajjad, Wang, & Ali, 2019).

Of the natural enemies reported in this study, the incidence of the hoverfly *P. borbonicus* was positively related to the densities of *L. e. pseudobrassicae* in the Coastal Savannah zone and *M. persicae* in the Deciduous Forest zone. Syrphids are among the most common predators associated with aphid infestations (Omkar, 2016). Previous studies have reported consistently high abundance of syrphid flies associated with aphid infestation on cabbage (Forchibe, Fening, & Afreh‐Nuamah, 2017; Fidelis et al., 2019; Fening et al., 2020), and a positive response by syrphids to increasing aphid densities (Honěk, 1983). Although spiders were positively related to *L. e. pseudobrassicae*, it should be noted that they are generalist predators, feeding on whatever prey that is present (Grez et al., 2014), and they were consistently recorded in this study, even in seasons with low or no aphid population. Coccinellids had a significant relationship with aphids in the Deciduous Forest agroecological zone, and none in the Coastal Savannah zone. Different species of coccinellids show different feeding habits (Fidelis et al., 2019), and thus could be responsible for the absence of any response in the Coastal Savannah zone. Additionally, though often associated with aphids, ladybugs are generalist predators (Snyder & Ives, 2003), and might have been preying on other pest species. On the other hand, Fidelis et al. (2018) reported coccinellidae larvae as one of the key mortality factors of *L. e. pseudobrassicae* and *M. persicae* on cabbage. Among the climatic variables, air temperature and rainfall affected the abundance of syrphids and spiders, while RH affected that of coccinellids. Rainfall and temperature have been reported to affect the population of spiders (Queiroz & Gasnier, 2017) and syrphids (Sajjad, Saeed, & Ashfaq, 2010).

In conclusion, *L. e. pseudobrassicae* had the highest population density throughout the study period compared to *M. persicae*, with both sometimes co‐occurring on cabbage. Their variation exhibits frequent peaks across different seasons and shows a vast difference between the two agroecological zones. Their densities are affected by plant phenology, weather conditions and predators, contributing largely to the observed variation trends. Furthermore, the major rainy season recorded the lowest densities of aphids in both agroecological zones, thus recommended for cultivation of cabbage due to low pest intensity. These findings provide key insights into the critical stage for aphid incidence and build‐up on cabbage, which should be further explored for timely and efficient management of these pests.

## AUTHOR CONTRIBUTIONS

**Ethelyn Echep Forchibe:** Conceptualization; data curation; formal analysis; funding acquisition; investigation; methodology; project administration; resources; validation; visualization; writing – original draft; writing – review and editing. **Ken Okwae Fening:** Conceptualization; funding acquisition; resources; supervision; validation; writing – review and editing. **Benjamin Narh‐Madey:** Formal analysis; software; validation; writing – original draft; writing – review and editing. **Kwame Afreh‐Nuamah:** Conceptualization; supervision; validation; writing – review and editing. **Millicent Cobblah:** Conceptualization; supervision; validation; writing – review and editing. **Francis Onono Wamonje:** Resources; supervision; validation; writing – review and editing. **John Peter Carr:** Funding acquisition; resources; supervision; validation; writing – review and editing.

## CONFLICT OF INTEREST STATEMENT

All authors declare that they have no conflicts of interest.

## Data Availability

The data that support the findings of this study are openly available in Zenodo, DOI: https://doi.org/10.5281/zenodo.7020754
